# Natural History of Leigh Syndrome: A Study of Disease Burden and Progression

**DOI:** 10.1002/ana.26260

**Published:** 2021-11-12

**Authors:** Albert Z. Lim, Yi Shiau Ng, Alasdair Blain, Cecilia Jiminez‐Moreno, Charlotte L. Alston, Victoria Nesbitt, Louise Simmons, Saikat Santra, Evangeline Wassmer, Emma L. Blakely, Doug M. Turnbull, Robert W. Taylor, Gráinne S. Gorman, Robert McFarland

**Affiliations:** ^1^ Wellcome Centre for Mitochondrial Research, Translational and Clinical Research Institute, Faculty of Medical Sciences Newcastle University Newcastle upon Tyne UK; ^2^ National Health Service Highly Specialised Services for Rare Mitochondrial Disorders Newcastle upon Tyne Hospitals National Health Service Foundation Trust Newcastle upon Tyne UK; ^3^ National Health Service Highly Specialised Services for Rare Mitochondrial Disorders Oxford University Hospitals National Health Service Foundation Trust Oxford UK; ^4^ Birmingham Children's Hospital Birmingham UK

## Abstract

**Objective:**

This observational cohort study aims to quantify disease burden over time, establish disease progression rates, and identify factors that may determine the disease course of Leigh syndrome.

**Methods:**

Seventy‐two Leigh syndrome children who completed the Newcastle Paediatric Mitochondrial Disease Scale (NPMDS) at baseline at 3.7 years (interquartile range [IQR] = 2.0–7.6) and follow‐up assessments at 7.5 years (IQR = 3.7–11.0) in clinics were enrolled. Eighty‐two percent of this cohort had a confirmed genetic diagnosis, with pathogenic variants in the *MT‐ATP6* and *SURF1* genes being the most common cause. The total NPMDS scores denoted mild (0–14), moderate (15–25), and severe (>25) disease burden. Detailed clinical, neuroradiological, and molecular genetic findings were also analyzed.

**Results:**

The median total NPMDS scores rose significantly (*Z* = −6.9, *p* < 0.001), and the percentage of children with severe disease burden doubled (22% → 42%) over 2.6 years of follow‐up. Poor function (especially mobility, self‐care, communication, feeding, and education) and extrapyramidal features contributed significantly to the disease burden (*τ*_b_ ≈ 0.45–0.68, *p* < 0.001). These children also deteriorated to wheelchair dependence (31% → 57%), exclusive enteral feeding (22% → 46%), and one‐to‐one assistance for self‐care (25% → 43%) during the study period. Twelve children (17%) died after their last NPMDS scores were recorded. These children had higher follow‐up NPMDS scores (disease burden; *p* < 0.001) and steeper increase in NPMDS score per annum (disease progression; *p* < 0.001). Other predictors of poor outcomes include *SURF1* gene variants (*p* < 0.001) and bilateral caudate changes on neuroimaging (*p* < 0.01).

**Interpretation:**

This study has objectively defined the disease burden and progression of Leigh syndrome. Our analysis has also uncovered potential influences on the trajectory of this neurodegenerative condition. ANN NEUROL 2022;91:117–130

Leigh syndrome is a genetically heterogeneous neurodegenerative disorder, typically characterized by stepwise developmental regression, symmetrical brainstem and/or basal ganglia involvement, and associated abnormal mitochondrial energy metabolism. Since this eponymous condition was first reported in 1951, it has become the commonest syndromic presentation of pediatric mitochondrial disease.^1^ The birth prevalence of Leigh syndrome is estimated to be 1 in 40,000^2^, ^3^ but rises to 1 in 2,000 in certain isolated populations.^4^, ^5^ Although our understanding of the clinical phenotype of Leigh syndrome has expanded rapidly by cross‐sectional studies,^6^, ^7^, ^8^, ^9^ it remains unclear how the natural history of this condition unfolds objectively in a longitudinal cohort study. Children who have this neurodegenerative condition are likely to accumulate disease burden over time. However, the exact rate of disease progression has not previously been determined. The dearth of robust data on the natural history of Leigh syndrome is hindering our understanding of disease mechanisms and factors influencing deterioration, as well as the design of interventional clinical trials in this condition. Despite the devastating nature of mitochondrial disease in children, the current clinical research landscape is underdeveloped when compared with that in adults. Therefore, we sought to quantify the disease burden and rate of progression of Leigh syndrome using a validated and established clinical scale, the Newcastle Paediatric Mitochondrial Disease Scale (NPMDS).^10^ This study also aims to identify factors that may determine disease trajectory and begins to address the void in systematic, purposeful natural history data collection. Data from this and subsequent studies will be crucial to the successful design of future clinical trials in mitochondrial disease.

## Patients and Methods

### Study Design and Participants

This observational longitudinal cohort study enrolled all children with confirmed diagnoses of Leigh syndrome who had completed 2 NPMDS assessments with expert clinicians. These children were part of the Mitochondrial Disease Patient Cohort (MitoCohort) UK: A Natural History Study and Patient Registry, which recruits individuals with a clinical diagnosis of mitochondrial disease, supported by evidence of a pathogenic variant(s) or a relevant biochemical deficiency (oxidative phosphorylation or pyruvate dehydrogenase). The NPMDS data were collected from the children who attended outpatient clinics in 2 UK hospitals (Newcastle and Birmingham) between March 2009 and March 2020. Clinicians at the Newcastle study site also reviewed children from Northern Ireland, Wales, and Scotland. The parents and legal guardians of participants provided written informed consent at enrollment into MitoCohort UK. No participants were deemed able to give consent, but age‐appropriate written information about the study was provided and assent was sought whenever possible. All parents of participants also consented to this study, which obtained a favorable opinion from an independent research ethics committee, NRES Committee North East (REC number: 13/NE/0326).

At the time of enrollment, these children were alive and were able to attend clinics at the study sites for assessments. Both baseline and follow‐up assessments were performed at their routine clinical outpatient appointments. All patients recruited to the study fulfilled the diagnostic criteria for Leigh syndrome^9^: (1) intellectual and motor developmental delay and/or regression; (2) clinical manifestation of corresponding symmetrical brainstem and/or basal ganglia neuroradiological changes; and (3) abnormal metabolism characterized by a defect in oxidative phosphorylation or pyruvate dehydrogenase complex activity, a molecular genetic diagnosis related to mitochondrial dysfunction, or elevated lactate in cerebrospinal fluid (CSF; >1.8mmol/l) or blood samples (>2.2mmol/l). Of those children with Leigh syndrome in the MitoCohort UK, 10 had not been eligible for the following reasons: had not completed 2 assessments (n = 5), Leigh‐like syndrome with subsequent alternative diagnoses (n = 3), and inadequate clinical data for follow‐up or analysis (n = 2).

### Outcome Measures

Our study used the NPMDS as the clinical outcome measure to determine objectively disease burden and progression over time.^10^ The scale has sections focusing on the current function (Section I), the system‐specific involvement (Section II), and the current clinical assessment (Section III). Each item has 4 responses: normal (0), and mild (1), moderate (2), and severe (3) impairment. The total score from all 3 sections, which reflects disease burden, can be categorized into mild (0–14), moderate (15–25), and severe (>25).^10^ The authors also critically reviewed the medical notes to reduce any potential information bias. The pediatricians (A.Z.L., E.W., V.N., and R.M.) conducting the NPMDS assessments worked independently of each other and were blinded to scores of any previous NPMDS assessments.

This study also collected other variables, including basic demographics, age at disease onset, weight centiles, and head circumference centiles. The Newcastle National Health Service Highly Specialised Service Mitochondrial Diagnostic Laboratory provided a molecular genetic diagnosis for all study participants. Analysis of the complete mitochondrial genome sequence was undertaken using Ion Torrent PGM^11^ or Sanger sequencing,^12^ as previously reported. Where no genetic defect was identified in mtDNA, nuclear genetic variants were identified using either candidate gene approaches (Sanger sequencing) or next generation sequencing including targeted panel and/or whole exome sequencing.^13^, ^14^ The other variable in this study was neuroimaging of these children. The whole brain magnetic resonance imaging (MRI; T1, T2, diffusion‐weighted imaging, and fluid‐attenuated inversion recovery sequences) was reviewed based on the diagnostic criteria for Leigh syndrome to include symmetrical lesions in the brainstem or basal ganglia structures, namely medulla, pons, midbrain, caudate, putamen, globus pallidus, and thalamus. Other areas of interest were also analyzed, including subcortical white matter, cerebral cortex, generalized atrophy, cerebellum, and corpus callosum. The neuroimaging investigation was performed at the time of clinical investigation for a diagnosis of Leigh syndrome.

### Statistical Analysis

SPSS v25 and R v4.0.3 were used for statistical analyses. All tests were 2‐sided, with significance set at *p* < 0.05. *T* tests were used for continuous variables and chi‐squared tests for categorical variables. To determine whether baseline NPMDS scores differed at follow‐up assessments, we used the Wilcoxon signed‐rank test and the Mann–Whitney *U* test. For the analysis of disease progression per annum, the change in NPMDS scores between baseline and follow‐up assessments was denominated by the years between assessments. Correlation coefficients were calculated with the appropriate measure (Pearson for continuous variables, Kendall tau‐b for ordinal variables). Logistic regression was used to determine factors that affected disease progression, with the probability of poor outcomes in patient subgroups estimated by Kaplan–Meier curves and log‐rank tests.

## Results

### Demographics

Seventy‐two children with Leigh syndrome from 68 different pedigrees fulfilled the inclusion criteria and were followed up over 2.6 years (standard deviation [SD] = 2.4, 95% confidence interval [CI] = 2.0–3.2). In this cohort, there were 34 boys and 38 girls. Twenty‐eight of these children (39%) have consanguineous parents. The median age at disease onset was 9 months (interquartile range [IQR] = 4.25–19). The median ages at their baseline and follow‐up NPMDS assessments were 3.7 years (IQR = 2.0–7.6) and 7.5 years (IQR = 3.7–11.0), respectively. The median weight of this group of children adjusted to their respective ages was on the 9th centile and the median head circumference was on the 2nd centile based on the UK World Health Organization growth reference charts.^15^ Many of these children took vitamin supplements either alone or in combination. These included ubiquinone (36%), riboflavin (17%), thiamine (11%), biotin (7%), folinic acid (5%), L‐carnitine (4%), and over‐the‐counter multivitamin drops (1%). None of these supplements was observed to influence the disease course during the follow‐up period of this study. Of the 6 children with evidence of pyruvate dehydrogenase deficiencies, 2 had ketogenic diet for epilepsy control, 2 had declined this treatment, and 2 had no clinical epilepsy to be considered eligible.

### Lactate and Respiratory Chain Enzyme Activities

Serum lactate levels were available for 50 children; 34 of them (68%) had higher levels than the standardized laboratory reference ranges (>2.2mmol/l). CSF lactate levels were measured in 36 children; 23 of them (64%) had high levels (>1.8mmol/l). Children who had abnormal CSF lactate levels did not differ significantly in disease burden (*Z* = −0.9, *p* = 0.365) or progression (*Z* = −0.3, *p* = 0.776). Thirty‐seven children had undergone skeletal muscle biopsy analysis at one of two national mitochondrial diagnostic laboratories in Newcastle and London. Of these, abnormal mitochondrial respiratory chain enzyme activities were reported in 18 cases (49%). The most common respiratory chain defect was an isolated complex I deficiency (n = 7), followed by an isolated complex IV deficiency (n = 6), followed by defects involving multiple oxidative phosphorylation components (n = 5). The finding of abnormal mitochondrial respiratory chain enzyme activities also did not differ significantly in disease burden (*Z* = −0.4, *p* = 0.713) or disease progression (*Z* = −0.1, *p* = 0.958).

### MRI Changes

Although all neuroimaging had been reported to fulfil the criteria of Leigh syndrome, 9 cranial MRI scans had to be excluded because of suboptimal quality for comparison and analysis. The neuroimaging for 63 children was available for analysis (53 alive and 10 who were deceased at the end of the study). Often these cranial MRI scans were performed at the time of initial investigation, at a mean age of 3.2 years (SD = 3.8, 95% CI = 2.0–4.4). Of those suitable for analysis, the most frequent finding was symmetrical putaminal signal abnormality (n = 36, 57.1%), followed by symmetrical changes in the globus pallidus (n = 26, 41.3%), and caudate (n = 25, 39.7%).

### Genotypic Spectrum

The genotypic spectrum of these 72 children is summarized in the Table 1. Fifty‐nine (82%) children in this cohort have a confirmed genetic diagnosis. Leigh syndrome secondary to pathogenic variants in the mitochondrial genome (GenBank accession: NC_012920.1) accounted for slightly less than one quarter of the cohort (n = 16, 22.2%). Pathogenic variants in the *MT‐ATP6* gene (m.8993 T > G, p.Leu156Arg, m.8993T>C, p.Leu156Pro and m.9176T>C, p.Leu217Pro) were the most common (n = 9) mtDNA etiologies in our Leigh syndrome cohort. Biallelic inherited variants accounted for most genetic diagnoses (n = 41, 56.9%), with defects in *SURF1* (NM_003172.4; n = 7) being the most common, followed by *NDUFV1* (NM_007103.4) (n = 5) and *ECHS1* (NM_004092.4; n = 5). Two children had X‐linked dominant inheritance of pathogenic variants in the *PDHA1* (NM_000284.4) gene. All pathogenic variants in this cohort have been classified according to the American College of Medical Genetics and Genomics guidelines,^16^ and this supplementary material is available online. Thirteen children had clinical and biochemical features consistent with a diagnosis of Leigh syndrome but remained genetically undetermined at the end of the study despite whole exome and mitochondrial genome sequencing.

**TABLE 1:** Genotypic Spectrum of the 72 Children with Leigh Syndrome from 68 Pedigrees in This Study

Inheritance	Mitochondrial Function	Gene	cDNA Change	Corresponding Protein Change	Children, n
Mitochondrial genome	Complex I subunits	*MT‐ND1*	m.3688G > A	p.Ala128Thr	1
		*MT‐ND4*	m.11778A > G	p.Arg340His	3
		*MT‐ND5*	m.12706 T > C, m.13513G > A	p.Phe124Leu, p.Asp393Asn	2
		*MT‐ND6*	m.14459G > A	p.Ala72Val	1
	Complex V subunits	*MT‐ATP6*	m.8993 T > C, m.8993 T > G, m.9035 T > C, m.9176 T > C	p.Leu156Arg, p.Leu156Pro, p.Leu170Pro, p.Leu217Pro	9
Nuclear genome	Complex I subunits	*NDUFV1*	c.1156C > T, c.1268C > T	p.Arg386Cys, p.Thr423Met	5
		*NDUFS1*	c.2102G > A, c.338 + 3A > G	p.Ser701Asn, p.Val88Glyfs*19	2
		*NDUFA9*	c.394C > T, c.1079G > A	p.Arg132*, p.Arg360His	1
	Complex I assembly factors	*NDUFAF6*	c.226 T > C	p.Ser76Pro	2
		*NDUFAF8*	c.45_52dup, c.195 + 271C > T	p.Phe18Serfs*32, splicing	1
	Complex IV assembly factors	*SURF1*	c.312‐321delinsAT, c.515 + 5G > C, c.792_793delAG, c.574_575insCTGC, c.752‐2A > G, c.488 T > G	p.Leu105*, splicing, p.Arg264Serfs*27, p.Arg192Profs*8, splicing, p.Val163Gly	7
	mtDNA maintenance and replication	*SUCLA2*	c.434C > A, c.272‐2A > C, c.1271del, c.1219C > T, c.851G > A	p.Thr145Lys, splicing, p.Gly424Aspfs*18, p.Arg407Trp, p.Arg284His	4
		*MPV17*	c.121C > T	p.Ser25Profs*49	1
	Mitochondrial translation factors	*MTFMT*	c.626C > T	p.Arg181Serfs*5	2
		*MTRFR*	c.96_99dup	p.Pro34Ilefs*25	1
		*TACO1*	c.460 T > C	p.Ser154Pro	2
	Mitochondrial fatty acid beta oxidation	*ECHS1*	c.251C > G, c.1A > T, c.518C > T, c.476A > G	p.Ala84Gly, start loss, p.Ala173Val, p.Gln159Arg	5
	Pyruvate dehydrogenase deficiency	*PDHA1*	c.759 + 26G > A, c.506C > T	p.Asp255Argfs*22, p.Ala169Val	2
		*PDHX*	c.1231C > T, c.1159C > T	p.Gln411*, p.Gln387*	3
	Aminoacyl‐tRNA synthetases	*NARS2*	c.670C > T, c.1142A > G	p.His224Tyr, p.Asn381Ser	2
		*DARS2*	c.228‐15C > A, c.492 + 2 T > C	p.Arg76Serfs*6, p.Met134_Lys165del	1
	Others	*BTD*	c.1241_1252del, c.1612C > T	p.Tyr414_Val417del, p.Arg538Cys	1
		*SLCA19A3*	c.1324_1327delinsAT	p.Tyr442Metfs*35	1
Others (presumed nuclear)	Isolated complex I deficiency				6
	Complex II + III deficiency				1
	Pyruvate dehydrogenase deficiency				1
	Persistently elevated lactate				5

### NPMDS: At Baseline and Follow‐Up

#### Section I: Current Function

**FIGURE 1:**
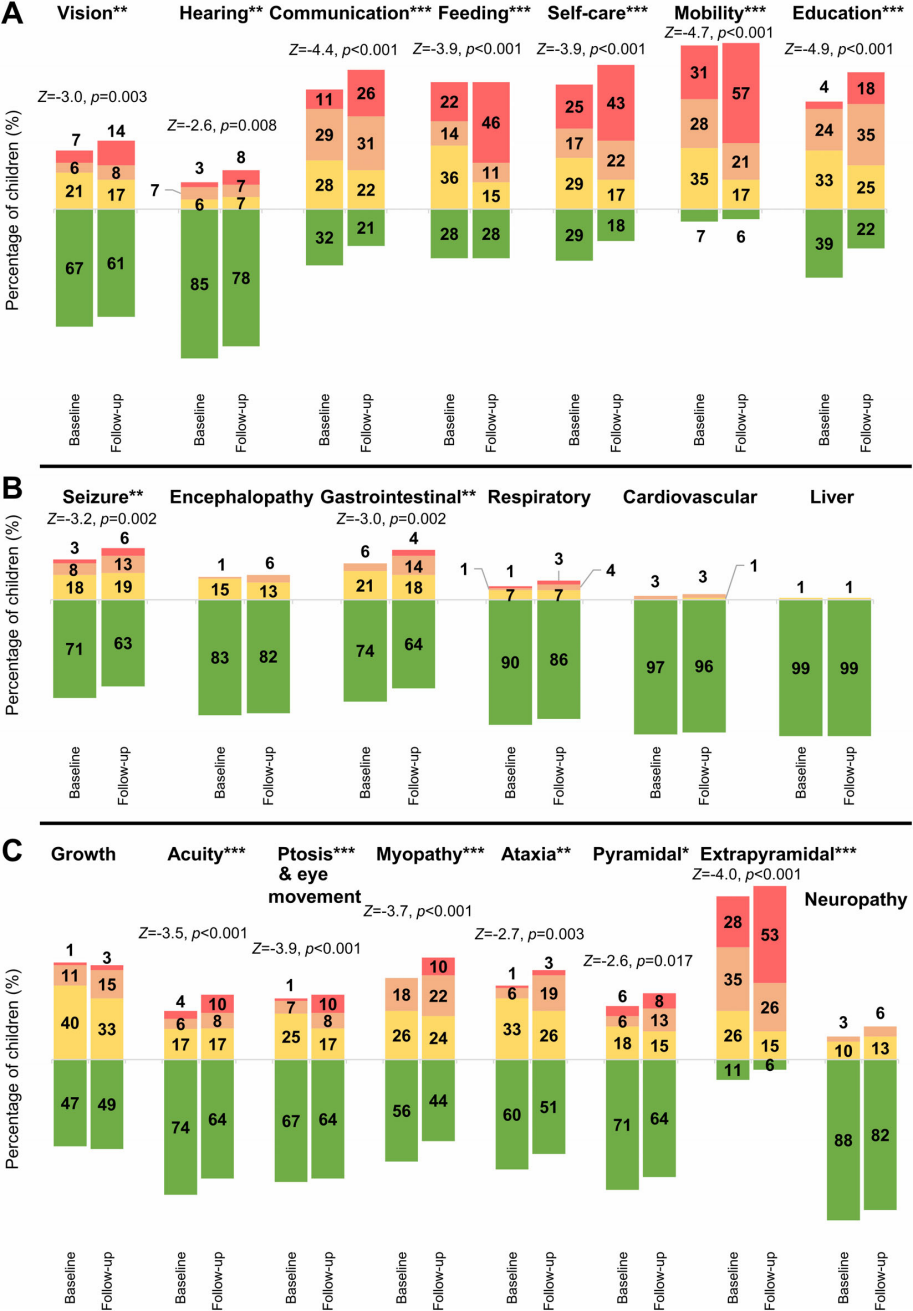
The Newcastle Paediatric Mitochondrial Disease Scale (NPMDS) ratings for each item in this cohort of children with Leigh syndrome. The percentages of children with Leigh syndrome were rated as normal (green), mild (yellow), moderate (amber), and severe (red) in selected items of the NPMDS. Baseline and follow‐up NPMDS scores were arranged alongside each other for comparisons in respective items: (A) current function, (B) system‐specific involvement, and (C) clinical assessment. *p < 0.05, **p < 0.01, ***p < 0.001. [Color figure can be viewed at www.annalsofneurology.org]

The proportion of children scoring mild, moderate, or severe disease ratings at their baseline and follow‐up NPMDS assessments is summarized in Figure 1. In the current function section (see Fig 1A), mobility was consistently reported as the most affected function in children with Leigh syndrome. Approximately one third of children (30.6%) were wheelchair‐dependent or fully reliant on their carer for mobility at baseline, but this increased significantly to 56.9% at follow‐up (*Z* = −4.7, *p* < 0.001). One quarter of children (25.0%) were fully reliant on parents with no contribution to self‐care at baseline; this rose significantly to 43.1% at follow‐up (*Z* = −3.9, *p* < 0.001). Although slightly more than one quarter of these children (27.8%) had normal feeding ability at baseline and follow‐up assessments, the proportion of children who had to be exclusively fed via gastrostomy or nasogastric tubes had doubled from 22.2% to 45.8% (*Z* = −3.9, *p* < 0.001). Similarly, the loss of ability to communicate effectively at an age‐appropriate level with parents doubled from 11.1% to 26.4% (*Z* = −4.4, *p* < 0.001). From their baseline to follow‐up assessments, children attending mainstream nursery or school with comparable academic achievement to peers dropped from 38.9% to 22.2% (*Z* = −4.9, *p* < 0.001). Approximately two thirds (66.7%) of these children had normal visual function without any concerns from parents or carers. At baseline, 5 children (6.9%) were registered as blind, or were using additional visual aids, or were unable to recognize faces, and this number had doubled to 10 children (13.9%) at follow‐up assessments (*Z* = −3.0, *p* = 0.03).

#### Section II: System‐Specific Involvement

Epileptic seizures and gastrointestinal systems were the two items in the System‐Specific Involvement section that changed significantly from baseline to follow‐up NPMDS assessments (see Fig 1B). The percentage of Leigh syndrome children with epileptic seizures had increased from 29.2% at baseline assessments to 37.5% at follow‐up assessments (*Z* = −3.2, *p* = 0.002). The other neurological manifestation of Leigh syndrome was encephalopathy; 16.7% and 18.1% of children had at least one episode in the preceding 6 months at baseline and follow‐up, respectively. Beyond the neurological features, gastrointestinal problems were the next most common system‐specific issue. One quarter (25.8%) of these patients had some forms of gut dysmotility symptom (NPMDS ≥ 1) at baseline assessment. At follow‐up NPMDS ratings, more than one third (36.2%) had gut‐related issues, with 4.2% having severe constipation with no relief from laxative treatment (*Z* = −3.0, *p* = 0.002). Cardiovascular (4.2%) and hepatic (1.4%) involvement were rare in our cohort of children with Leigh syndrome. No children had endocrine, renal, or hematological system involvement.

#### Section III: Current Clinical Assessment

The baseline and follow‐up clinical assessments have been summarized in Figure 1C. Extrapyramidal signs were the most common examination finding, identified in 88.9% and 94.4% of children at baseline and follow‐up assessments, respectively. At baseline clinic visit, 27.8% of children in this cohort had severe extrapyramidal disorders, characterized by dystonia and dyskinesia, which resulted in wheelchair dependency. The percentage of children with severe extrapyramidal features rose at their follow‐up to 52.8% (*Z* = −4.0, *p* < 0.001). Likewise, the percentage of children with muscle weakness or myopathy had increased from baseline (*Z* = −3.7, *p* < 0.001). Cerebellar ataxia also increased from baseline (*Z* = −2.7, *p* = 0.003), with 19.4% of children needing assistance with gait abnormality or severe limb dysmetria at follow‐up. Another neurological symptom that worsened significantly was pyramidal signs (*Z* = −2.6, *p* = 0.017). Deterioration of visual acuity and ptosis had also increased from their initial assessments (*Z* = −3.5, *p* < 0.001). The severity of neuropathy did not change (*Z* = −1.4, *p* = 0.177). Although the developmental scores had not differed significantly between the two assessments (*Z* = −0.01, *p* = 0.991), almost all of these children had some degree of developmental delay, with nearly one third (29.2%) having severe developmental regression in the 4 months preceding their follow‐up assessments.

### Interitem Relationships

**FIGURE 2:**
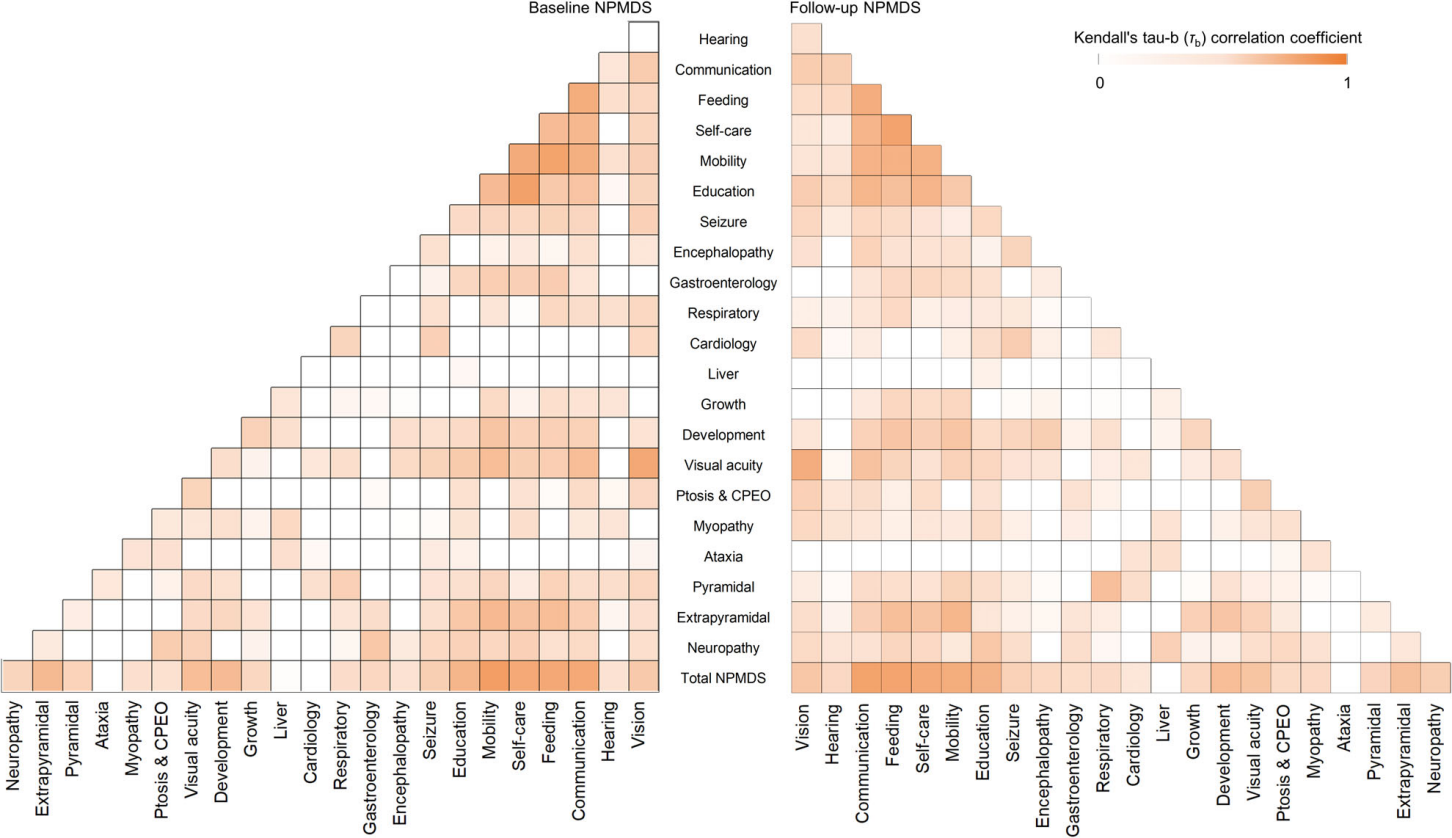
The interitem relationships of the Newcastle Paediatric Mitochondrial Disease Scale (NPMDS) items in this cohort of children with Leigh syndrome. Any positive Kendall tau‐b (*τ*_b_) correlation coefficients are shown in darker shades. The correlation matrix at baseline (left) and at follow‐up assessments (right) were similar. CPEO = chronic progressive external ophthalmoplegia. [Color figure can be viewed at www.annalsofneurology.org]

The clinical manifestation of these children with Leigh syndrome can affect several interrelated items within the NPMDS, and these items can contribute differently to the total scores. Some of these items have been shown to be correlated with each other (Fig 2). In Section 1 of the NPMDS, which indicates the functional status of the child, feeding, self‐care, communication, and mobility were all strongly correlated (*τ*_b_ ≈ 0.53–0.67, *p* < 0.001). The educational attainment of these children was also correlated with the ability to self‐care (*τ*_b_ ≈ 0.52, *p* < 0.001) and communicate (*τ*_b_ ≈ 0.51, *p* < 0.001) appropriately for their respective ages. The scores on these 5 items (communication, feeding, self‐care, mobility, and education) within the functional status of the children strongly correlated with the total NPMDS scores (*τ*_b_ ≈ 0.53–0.68, *p* < 0.001). In the clinical assessment section, extrapyramidal signs showed a correlation with mobility at both baseline (*τ*_b_ ≈ 0.46, *p* < 0.001) and follow‐up assessments (*τ*_b_ ≈ 0.50, *p* < 0.001). The correlation between extrapyramidal signs and the total NPMDS scores was also significant (*τ*_b_ ≈ 0.45, *p* < 0.001).

### Disease Burden and Progression

**FIGURE 3:**
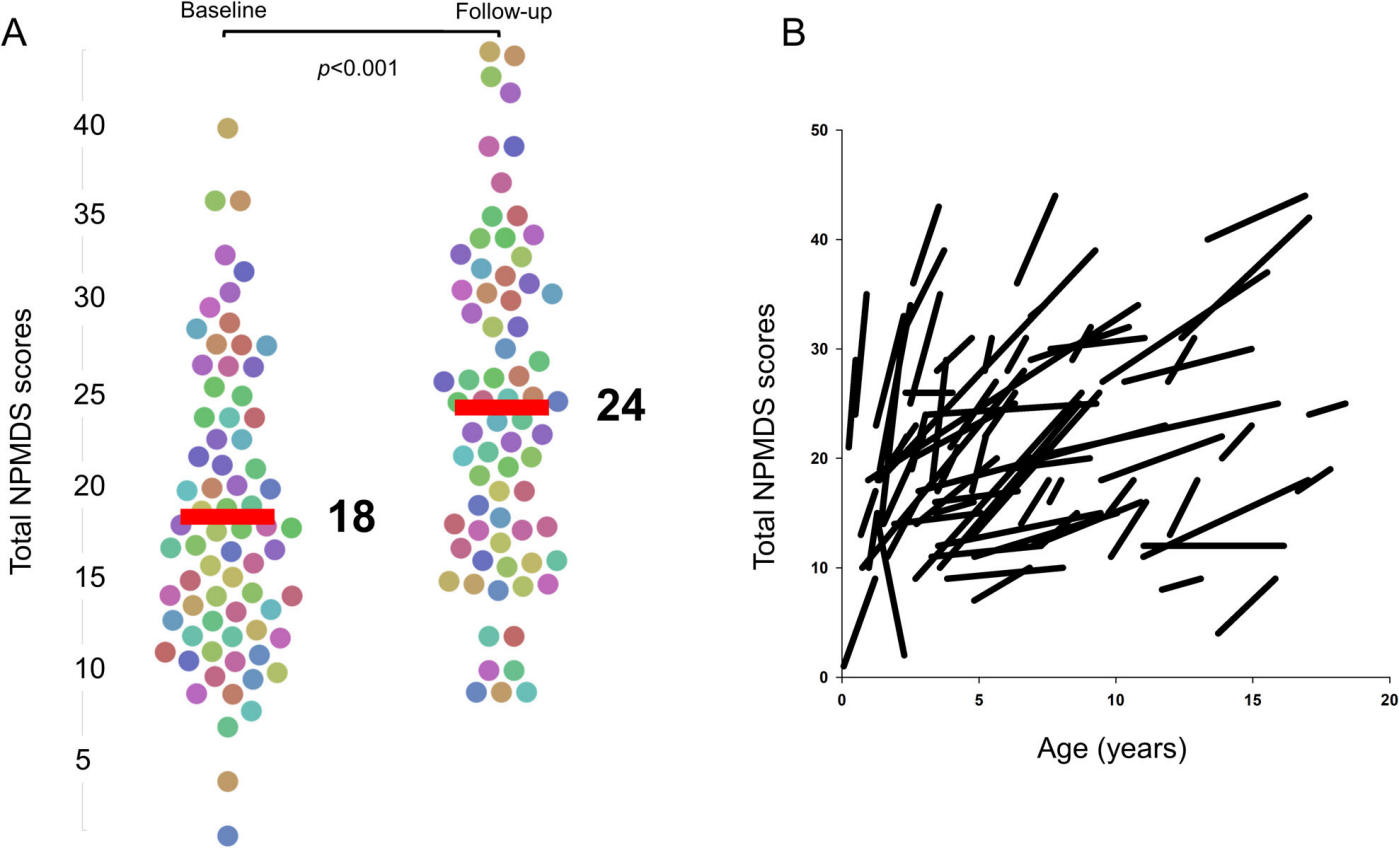
The change of total Newcastle Paediatric Mitochondrial Disease Scale (NPMDS) scores for all participants. (A) A bee swarm plot of the total NPMDS scores for each individual patient in this study at baseline (left swarm) and at follow‐up (right swarm). Horizontal bars indicate the median scores. The median score rose from 18 at baseline to 24 at follow‐up NPMDS assessments. The difference between these two assessments was significant at *p* < 0.001. (B) A vector graph showing these changes according to their ages. Each individual line shows the change in NPMDS scores from baseline to follow‐up assessments for every patient in this study. [Color figure can be viewed at www.annalsofneurology.org]

The median NPMDS scores at baseline and follow‐up assessments were 18 (IQR = 12–24) and 24 (IQR = 17–31), respectively (Fig 3A). A Wilcoxon signed‐rank test confirmed that this change in NPMDS scores was significant (*Z* = −6.9, *p* < 0.001). Almost all of the children experienced an increase in the NPMDS score from their respective baseline assessments (see Fig 3B) except for one child who had a diagnosis of biotinidase deficiency (biallelic pathogenic *BTD* gene variants) and improved clinically with the administration of high‐dose biotin. This improvement was evident by the fall in the total NPMDS score from 14 to 2. At their respective baselines, only 16 children (22.2%) had a high disease burden, with an NPMDS score > 25. The number of children with the high disease burden nearly doubled to 30 (41.7%) at their respective follow‐up.

**FIGURE 4:**
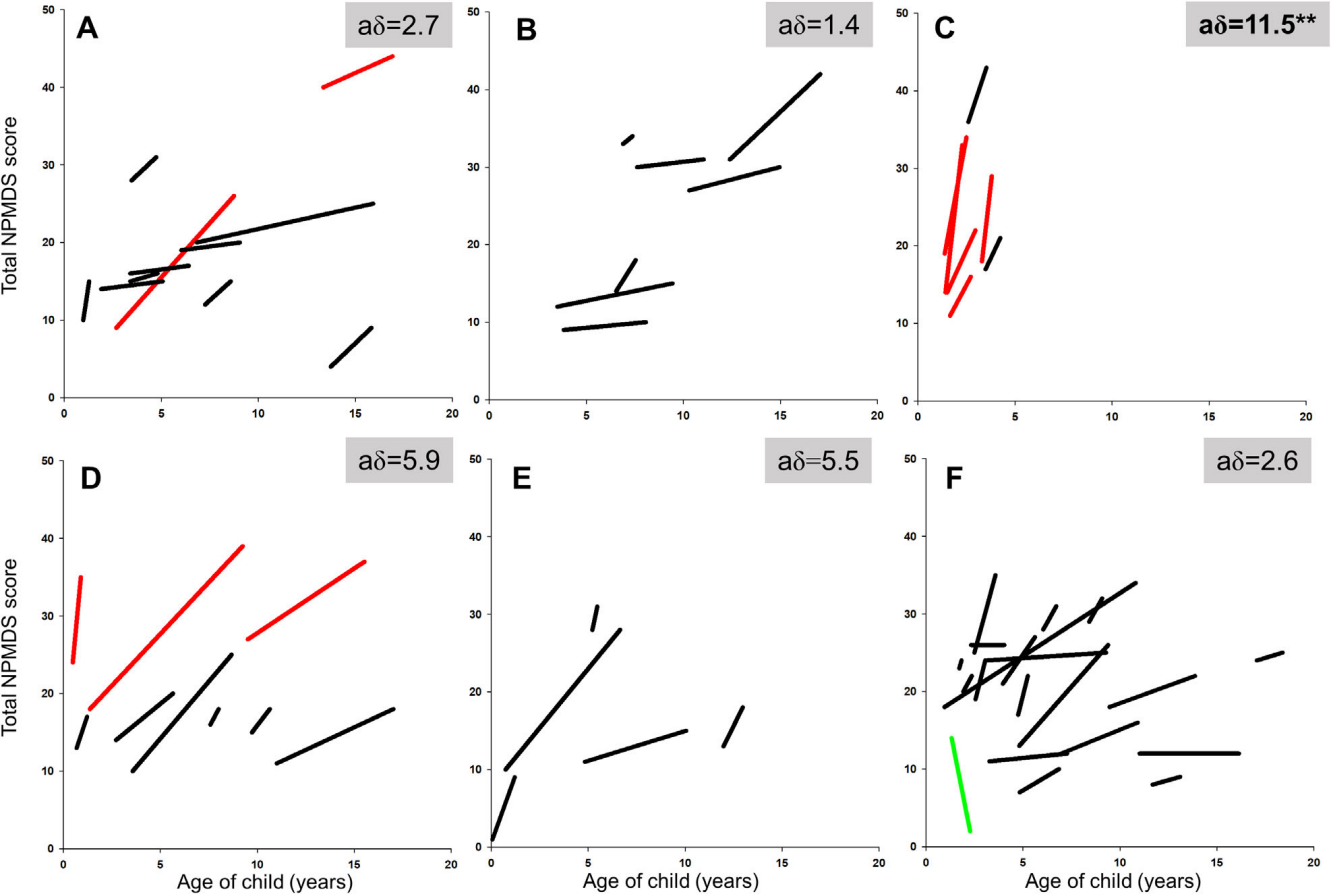
The vector plots for Newcastle Paediatric Mitochondrial Disease Scale (NPMDS) scores at baseline and at follow‐up in this study based on their genotypes. The grey vector lines indicate patients who had died since the end of study. aδ represents the change of NPMDS scores per annum. Vector plots are categorized into several groups according to genotypes: (A) complex I assembly factor and structural protein variants (*NDUFV1*, *NDUFS1*, *NDUFA9*, *NDUFA13*, *NDUFAF6*, *NDUFAF8*), (B) mtDNA‐encoded complex I subunits variants (*MT‐ND1*, *MT‐ND4*, *MT‐ND5*, *MT‐ND6*), (C) pathogenic *SURF1* gene variants affecting complex IV assembly, (D) *MT‐ATP6* gene variants, (E) pathogenic variants affecting pyruvate dehydrogenase complex (*PDHA1*, *PDHX*), and (F) other pathogenic nuclear gene variants. Children without a known genetic diagnosis are not shown here. **p < 0.01. [Color figure can be viewed at www.annalsofneurology.org]

The children in this cohort gained on average 4.5 points on the NPMDS score annually (SD = 6.5, 95% CI = 3.0–6.1). This increment of NPMDS score per annum differed among genotypes (Fig 4). Patients who harbored pathogenic *SURF1* variants (n = 7), impairing the assembly of complex IV, had the highest rise in NPMDS score per annum at 11.5 (SD = 7.7, 95% CI = 4.3–18.5). This was followed by patients harboring pathogenic *MT‐ATP6* gene variants encoding a structural subunit of complex V (5.9; SD = 8.1, 95% CI = 0–12.1), pathogenic variants in *PDHA1* and *PDHX*, genes causing pyruvate dehydrogenase deficiency (5.5; SD = 4.2, 95% CI = 0.3–10.8), variants in nuclear genes affecting complex I assembly proteins and structural subunits (2.7; SD = 4.6, 95% CI = ‐0.4 to 5.8), other nuclear genotypes (2.6; SD = 5.0, 95% CI = 0.3–5.0), and pathogenic variants affecting mtDNA‐encoded complex I subunits (1.4; SD = 1.4, 95% CI = 0.1–2.7). Compared to the other genotypes, the annual NPMDS score increment in patients with pathogenic *SURF1* variants was significantly higher, *t*(70) = 3.1, *p* = 0.002.

### Survival Status

Twelve children (16.6%) died during the follow‐up period. The median age at death was 4.0 years (IQR = 2.7–12.9) with a mean of 7.3 years (SD = 6.0, 95% CI = 3.5–11.1). The mean interval between their last follow‐up NPMDS and their death was 8.7 months (SD = 8.7, 95% CI = 3.1–14.3). Among all the domains within the follow‐up NPMDS, the deceased group scored significantly higher on the following items: feeding function (*Z* = ‐2.5, *p* = 0.013), respiratory involvement (*Z* = ‐4.9, *p* < 0.001), and pyramidal assessment (*Z* = ‐2.4, *p* = 0.015).

### Possible Predictors of Outcomes

#### Early Age at Onset

**FIGURE 5:**
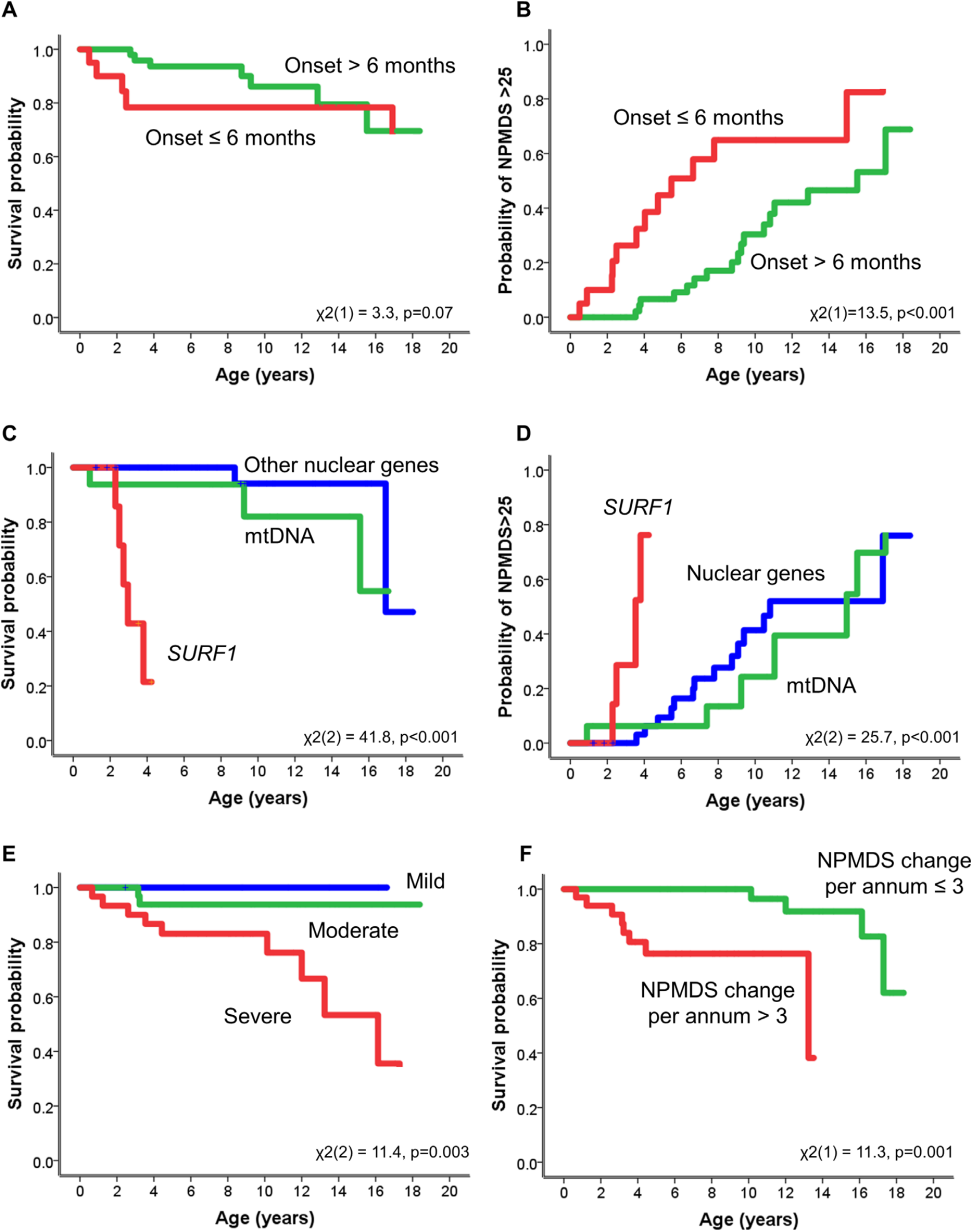
The Kaplan–Meier curves for survival probability from mortality and from severe disease burden (Newcastle Paediatric Mitochondrial Disease Scale [NPMDS] score > 25). (A) Survival probability for those children with disease onset at 6 months and younger. (B) Probability of NPMDS > 25 for disease onset at 6 months and younger. (C, D) Probability of *SURF1* cases compared to other nuclear and mitochondrial pathogenic variants from mortality (C) and severe disease burden (NPMDS > 25; D). There were significant differences between the *SURF1* cases and the other two genotypes. (E) The survival probability of different disease burden based on NPMDS scores (mild, 0–14; moderate, 15–25; severe, >25). Those children with severe disease burden (NPMDS > 25) had significantly worse survival than those who had mild or moderate disease burden. (F) The survival probability of disease progression shows that those who had a change of >3 points on the NPMDS scores per annum also had significantly poorer survival outcomes. [Color figure can be viewed at www.annalsofneurology.org]

Although the surviving children had later age at disease onset (mean = 16.2 months, SD = 17.9, 95% CI = 11.6–20.8) than those who died (mean = 12.2 months, SD = 13.0, 95% CI = 3.9–20.4), this difference was not significant (*t*[70] = −0.744, *p* = 0.459). In this cohort, 20 children (27.7%) had their onset of disease before the age of 6 months. Early disease onset (<6 months) was correlated with a significantly higher NPMDS score at follow‐up assessments (*U* = 339.5, *p* = 0.022). The log‐rank test of differences in the survival of children with disease onset at <6 months and those with later disease onset was not statistically significant (χ^2^[1] = 3.3, *p* = 0.07; Fig 5A). However, the probability of high disease burden (NPMDS > 25) between these two groups was significant (χ^2^[1] = 13.5, *p* < 0.001; see Fig 5B).

#### Genotypes

The 12 children who had died after their last follow‐up assessment harbored pathogenic variants in the following genes: *SURF1* (n = 5), *MT‐ATP6* (n = 3), *NUDFAF6* (n = 1), *NDUFS1* (n = 1), and genetically undetermined (n = 2). In comparison to pathogenic gene variants in nuclear or mtDNA, children with Leigh syndrome as a result of pathogenic recessive variants in the *SURF1* gene were significantly associated with poorer survival (χ^2^[2] = 41.8, *p* < 0.001; see Fig 5C) and were associated with a 3.1‐fold increased risk of death (risk ratio = 3.1, 95% CI = 1.0–10.1). The difference in survival in those with high disease burden (NPMDS > 25) was also statistically significant (χ^2^[2] = 25.7, *p* < 0.001), and again children with Leigh syndrome due to recessive *SURF1* pathogenic variants fared worse (see Fig 5D). The main pathogenic *SURF1* variants identified were c.312‐321del10insAT, p.Leu105*, c.792_793delAG, p.Arg264Serfs*27, and c.574_575insCTGC, p.Arg192Profs8* variants.

#### Disease Burden and Progression

Children who subsequently died had a higher follow‐up NPMDS median score of 32 (IQR = 26.75–36.5) than those who survived, where the median score was 22.5 (IQR = 16–29.5; *Z* = −3.0, *p* < 0.01). However, there was no difference in the baseline NPMDS scores for the surviving and the deceased groups, with medians of 17 (IQR = 12–24) and 18.5 (IQR = 14–26.25), respectively (*Z* = ‐0.6, *p* = 0.562). Children with severe disease burden (total NPMDS score > 25) had higher mortality than those with lower burden (χ^2^[2] = 11.4, *p* = 0.003; see Fig 5E). Having severe disease burden was still significantly associated with mortality even after controlling for the other independent variables sex, age at onset, and genotype (*p* < 0.01). Another indicator of poor survival is the change in NPMDS per annum. Children who died exhibited significantly faster disease progression prior to their death, as evidenced by the higher acquisition of annual NPMDS scores per annum (11.5; SD = 11.0, 95% CI = 4.5–18.6) than those who remained alive (3.2; SD = 4.0, 95% CI = 2.1–4.2; *t*[70] = 4.6, *p* < 0.001). Those children whose NPMDS scores rose by >3 points per annum had significantly worse survival in the log‐rank test of differences (χ^2^[1] = 11.3, *p* = 0.001; see Fig 5F).

#### Neuroimaging Changes

**FIGURE 6:**
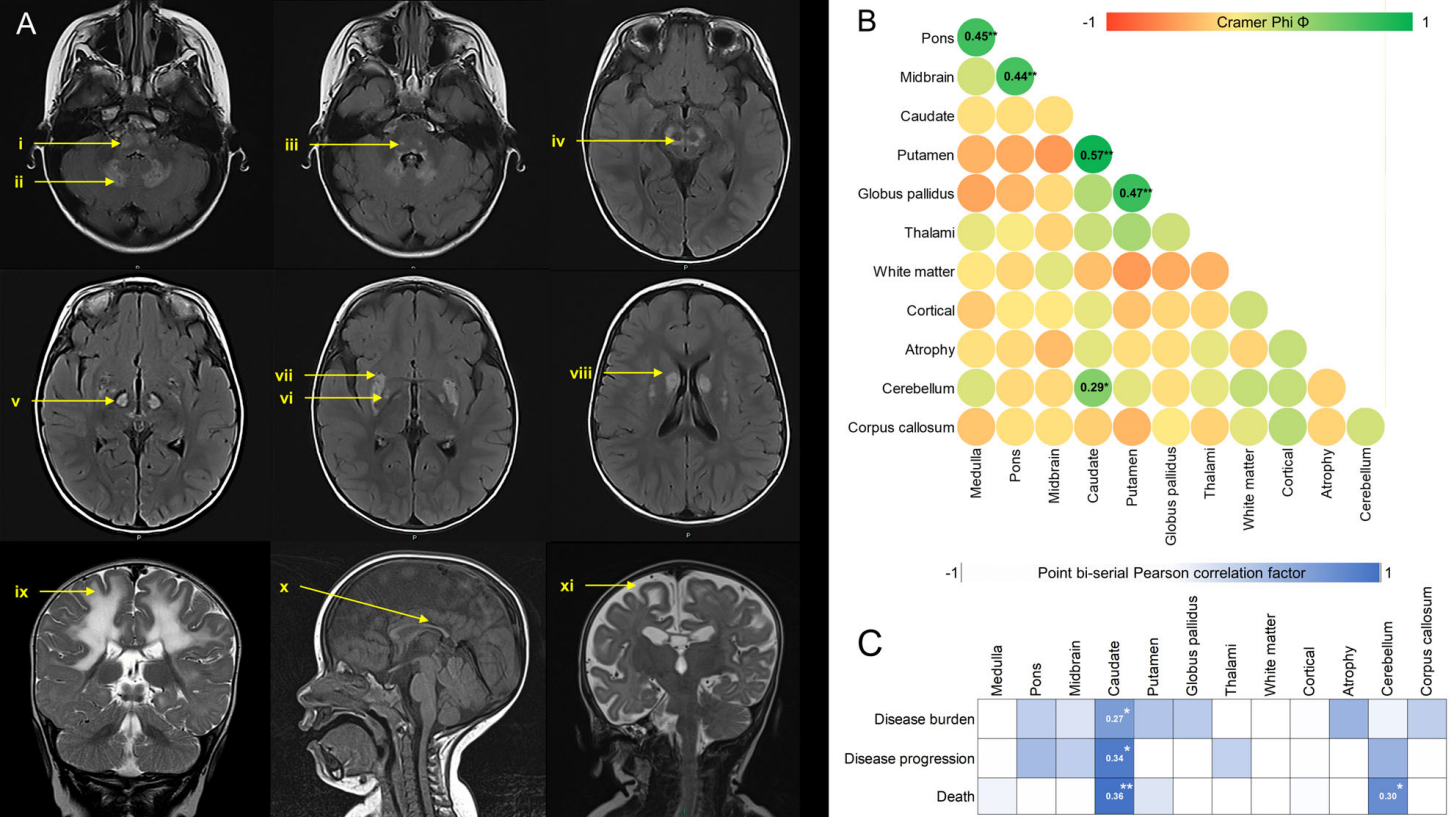
Neuroimaging of this cohort of children with Leigh syndrome. (A) A representative neuroimaging changes in these children with Leigh syndrome: (i) medulla, (ii) cerebellar nuclei, (iii) pons, (iv) midbrain, (v) thalami, (vi) globus pallidus, (vii) putamen, (viii) caudate, (ix) white matter involvement, (x) thin corpus callosum, (xi) generalized atrophy. (B) The correlation matrix of Φ values of positive findings for each region of the brain based on the neuroimaging images of 63 patients. Green represents positive nominal‐by‐nominal association. (C) The point biserial Pearson correlation factor for each abnormal neuroimaging change against the disease burden (follow‐up Newcastle Paediatric Mitochondrial Disease Scale [NPMDS] scores), disease progression (NPMDS score change per annum), and death. Blue indicates positive correlation. *p < 0.05, **p < 0.01. [Color figure can be viewed at www.annalsofneurology.org]

Representative neuroimages from this cohort are summarized in Figure 6A. The basal ganglia structures, especially the dorsal striatum (caudate nuclei and putamen), are often observed to be involved together on MRI (see Fig 6B). Bilateral caudate nuclei signal abnormality is also positively correlated with higher follow‐up NPMDS score (*r*_pb_ = 0.27, *p* = 0.033), incremental change when assessed per annum (*r*_pb_ = 0.34, *p* = 0.007), and mortality (*r*_pb_ = 0.36, *p* = 0.004; see Fig 6C). Bilateral cerebellar signal changes were identified in 30% of those who died compared with 5.7% of those who were still alive (χ[1] = 5.8, *p* = 0.046). Generalized brain atrophy at initial neuroimaging is associated with higher NPMDS scores at follow‐up assessments (χ[27] = 47.5, *p* = 0.009).

## Discussion

This cohort study has provided new natural history data to define the clinical evolution of Leigh syndrome. This group of children have been recruited over the past 12 years within MitoCohort UK, the largest cohort of patients with mitochondrial disease in the UK. This longstanding recruitment has been delivered by national centers of excellence in the diagnosis and clinical management of patients with mitochondrial disease, where specialist diagnostic laboratories have provided a genetic diagnosis in >80% of these children, in contrast to much lower yields of genetically confirmed cases in other cohorts.^8^, ^17^, ^18^, ^19^ Another major advantage of this longitudinal study in Leigh syndrome over previously published data from cross‐sectional observational studies is the systematic chronological recording of a validated mitochondrial disease–specific rating scale (NPMDS)^10^, ^20^ that documented and quantified clinical changes in individual patients. The NPMDS assessment tool was applied by an expert group of clinicians who have been trained in its administration. Repeated use of the NPMDS at multiple outpatient clinic attendances not only provides quantitative data on the disease severity of Leigh syndrome but also allows disease trajectories to be plotted for individual patients. Furthermore, the NPMDS has several subsections that can afford detailed scrutiny of subtle phenotypic variations arising from the diverse genotypic etiology of Leigh syndrome.

Our intention to define the natural history of Leigh syndrome in a way that is suitable for interventional studies in outpatient settings has led to some limitations. We observed 12 deceased children (16.6%) in this cohort, contrary to the higher mortality of Leigh syndrome reported by previous cross‐sectional studies.^2^, ^8^, ^19^, ^21^ This higher survival rate could be attributed to the design of this study, which focused on children who have likely already survived acute crises in their early years. Early phase clinical trials in rare diseases are fraught with logistical challenges, not least of which are establishing the genetic diagnosis, homogeneity of phenotype, natural history, and time to recruitment.^22^, ^23^, ^24^ This cohort of genetically confirmed Leigh syndrome patients with a slower disease course and longer survival are probably best placed for recruitment to such early phase clinical trials. Those children who had rapid deterioration in very early life might experience only a narrow window of opportunity for enrollment in early phase trials.^25^ Furthermore, early case reports of genetic conditions are often descriptions of strikingly severe or novel disease manifestations, but almost inevitably these underestimate the breadth of phenotype associated with any given genetic defect. This is true for Leigh syndrome, and the less severe phenotypes that we have observed in this cohort contrast with those previously published and probably contribute to the survivor bias. Disease onset before 6 months of age has been a poor prognostic factor in some studies.^8^, ^19^ In this study, early disease onset is not a predictor of mortality but is associated with a higher probability of developing severe disease burden (NPMDS score > 25). However, the relatively small number, especially of rare pathogenic variants, means that it is not possible to draw valid comparisons. Another limitation of our study is the use of only 2 time points. For some children with Leigh syndrome, this approach may have missed a stepwise deterioration and erroneously abbreviated their disease course to a linear decline. In addition, through analysis of the NPMDS data, we have observed a ceiling effect from the ordinal scoring of certain items in the scale, which can substantially underestimate the “real life” burden of Leigh syndrome.

Notwithstanding the limitations, this study has uncovered several key findings for the mitochondrial community. First, this cohort of 72 children has encountered a considerable disease burden and disease progression. The current function section (Section 1) of the NPMDS highlighted the worsening of the severity on all items, particularly mobility, education, communication, and self‐feeding skills. Each of these functional activities was interrelated and contributed significantly to the overall disease burden. Most notably, more than half of these children progressed to full wheelchair or carer dependence for their mobility at follow‐up assessments. The loss of mobility is an established risk for increased mortality in the pediatric population.^26^ The impact of this significant change in daily function was also evident in the other items of the NPMDS. Of the items in the NPMDS, the progression of extrapyramidal features was the most prominent. More importantly, children with severe disease burden indicated by NPMDS scores > 25 and those shown to have faster disease progression by accumulation of >3 points in the NPMDS score per annum are more likely to have poorer survival.

Apart from disease burden and progression, there are also other potential predictors of poorer outcomes in these children. Children with Leigh syndrome due to pathogenic *SURF1* variants in this cohort have poorer outcomes when followed up longitudinally. The *SURF1* gene encodes for an assembly factor of mitochondrial cytochrome *c* oxidase.^27^ Children in this cohort with SURF1 deficiency have rapid disease progression and high mortality as compared to other genotypes. This poor prognosis is in contrast to a published cross‐sectional study of patients with SURF1 deficiency.^28^ The median age at death of our Leigh syndrome participants with pathogenic *SURF1* variants is lower than that observed from the cross‐sectional study,^28^ which had different inclusion criteria, resulting in a broader spectrum of phenotypes being recruited. Therefore, it is difficult to compare these two studies directly with regard to survival.

Another interesting finding from this study is the association of caudate involvement with disease burden, progression, and mortality. The majority of patients in this study have striatal changes (putamen and caudate) within the basal ganglia, and this is similarly reported in the literature.^29^, ^30^, ^31^ Often these basal ganglia structures were involved together, probably as a consequence of their close anatomical and physiological integration.^32^, ^33^ In a *Ndufs4* knockout mouse model of Leigh syndrome and complex I deficiency, the selective inactivation of these striatal neurons led to progressive motor impairment.^34^ In human studies, caudate lesions are often associated with neurobehavioral changes and progressive cognitive decline in adults,^35^, ^36^, ^37^ but this phenomenon in children remains unclear. It is not possible to postulate from the findings of this study how the caudate nuclei lesions disrupt the neural network to other cerebral structures in Leigh syndrome, but this link is worth exploring in future histopathological studies. Alternatively, the presence of caudate lesions could partly be a result of advanced stage of the disease process in Leigh syndrome. The caudate lesions in this cohort of children with Leigh syndrome are not seen in isolation but in conjunction with other accompanying symmetrical lesions in the basal ganglia or brainstem.

From a clinical perspective, this study has illustrated that the use of this objective scale in clinics for patients with Leigh syndrome is of value for monitoring, prognostication, and advance care planning. Given the growing landscape for therapeutic intervention in mitochondrial diseases, this study will also be of substantial value to the design of future trials as a historical metric of standard clinical care for those where a control arm might not be feasible. This study also highlights the need to set up large‐scale international collaborative studies that recruit more patients, especially those with rarer genotypes, to explore a range of sensitive outcome measures alongside NPMDS in a longitudinal assessment of these patients.

## Conclusions

We have established objective information on the disease burden and progression of Leigh syndrome caused by different genotypes. Furthermore, we have identified several predictors of disease trajectory in this devastating neurodegenerative condition.

## Author Contributions

A.Z.L., Y.S.N., G.S.G., and R.M. contributed to the conception and design of the study. A.Z.L., Y.S.N., A.B., C.J.‐M., V.N., L.S., S.S., E.W., C.L.A., E.L.B., R.W.T., and R.M. contributed to the acquisition and analysis of data. A.Z.L., Y.S.N., A.B., C.L.A., D.M.T., R.W.T., G.S.G., and R.M. contributed to drafting the text and preparing the figures.

## Potential Conflicts of Interest

Nothing to report.

## Supporting information


**TABLE S1**. Supporting information.
