# Supplementary material for: Natural History of Leigh Syndrome: A Study of Disease Burden and Progression
Source: Ann Neurol. 2021 Nov 12;91(1):117–30. doi: 10.1002/ana.26260 (PMC9534328; doi:10.1002/ana.26260)
Supplement: Supplementary file 1 — TABLE S1. Supporting information. [file ANA-91-117-s001.docx]

**Supplementary Table 1**

| **Gene** | **RefSeq** | **cDNA** | **Protein** | **Pathogenicity** | **Criteria [ACGS 2020 guidelines]^1^** | **References** |
| --- | --- | --- | --- | --- | --- | --- |
| *BTD* | NM_000060.4 | c.1241_1252del | p.Tyr414_Val417del | Pathogenic | PS3_mod; PM2_mod; PM3_vstr; PM4_mod; PP1_mod; PP4_sup | Pomponio et al 1997^2^ |
| *BTD* | NM_000060.4 | c.1612C>T | p.Arg538Cys | Pathogenic | PS3_mod; PM2_mod; PM3_vstr; PM5_sup; PP1_str; PP3_sup | Pomponio et al 1997^2^; Wiltink et al 2016^3^ |
| *MTRFR* | NM_152269.5 | c.96_99dup | p.Pro34Ilefs*25 | Pathogenic | PVS1_vstr; PM2_mod; PM3_str; PP1_str | Heidary et al 2014^4^ |
| *DARS2* | NM_018122.5 | c.228n.d.15C>A | p.Arg76Serfs*6 | Pathogenic | PM2_mod; PM3_vstr; PS3_sup | Scheper 2007^5^; Van Berge et al 2014^6^ |
| *DARS2* | NM_018122.5 | c.492+2T>C | p.Met134_Lys165del | Pathogenic | PM2_mod; PM3_vstr; PS3_sup | Scheper et al 2007^5^; Stellingwerff et al 2021^7^ |
| *ECHS1* | NM_004092.4 | c.251C>G | p.Ala84Gly | Likely Pathogenic | PM2_mod; PM3_sup; PP1_sup | In-house analysis |
| *ECHS1* | NM_004092.4 | c.1A>T | start loss | Likely Pathogenic | PVS1_mod; PS1_mod; PM2_mod | Genomics England 100K project |
| *ECHS1* | NM_004092.4 | c.518C>T | p.Ala173Val | Likely Pathogenic | PM2_mod; PM3_str; PP4_sup | Mahajan et al 2017^8^; Olgiati et al 2016^9^ |
| *ECHS1* | NM_004092.4 | c.476A>G | p.Gln159Arg | Pathogenic | PS3_mod; PM2_mod; PM3_vstr; PP1_str; PP4_sup | Haack et al 2015^10^ |
| *MPV17* | NM_002437.5 | c.121C>T | p.Ser25Profs*49 | Pathogenic | PS3_str; PM2_mod; PM3_str; PM3_mod; PP1_sup; PP3_sup | In-house analysis |
| *MTFMT* | NM_139242.4 | c.626C>T | p.Arg181Serfs*5 | Pathogenic | PS3_str; PM2_mod; PM3_vstr; PP3_sup | Tucker et al 2011^11^ |
| *NARS2* | NM_024678.6 | c.670C>T | p.His224Tyr | Likely Pathogenic | PS3_sup; PM2_mod; PM3_mod; PP3_sup; PP4_sup | NOVEL VARIANT |
| *NARS2* | NM_024678.6 | c.1142A>G | p.Asn381Ser | Pathogenic | PM2_mod; PM3_str; PP1_str; PP4_sup | Simon et al 2015^12^ |
| *NDUFA9* | NM_005002.5 | c.394C>T | p.Arg132* | Pathogenic | PVS1_vstr; PM2_mod | NOVEL VARIANT |
| *NDUFA9* | NM_005002.5 | c.1079G>A | p.Arg360His | Likely Pathogenic | PM2_mod; PM3_mod; PM5_sup; PP3_sup | NOVEL VARIANT |
| *NDUFAF6* | NM_152416.4 | c.226T>C | p.Ser76Pro | Pathogenic | PS3_str; PM_mod; PM3_str; PP1_mod | Kohda et al 2016^13^ |
| *NDUFAF8* | NM_001086521.1 | c.45_52dup | p.Phe18Serfs*32 | Pathogenic | PVS1_str; PS3_str; PM2_mod | Alston et al 2020^14^ |
| *NDUFAF8* | NM_001086521.1 | c.195+271C>T | splicing | Likely Pathogenic | PS3_str; PM2_mod; PM3_mod | Alston et al 2020^14^ |
| *NDUFS1* | NM_005006.7 | c.2102G>A | p.Ser701Asn | Likely Pathogenic | PM2_mod; PM3_mod; PP3_sup; PP4_sup | In-house analysis |
| *NDUFS1* | NM_005006.7 | c.338+3A>G | p.Val88Glyfs*19 | Likely Pathogenic | PS3_mod; PM2_mod; PM3_sup; PP4_sup | NOVEL VARIANT |
| *NDUFV1* | NM_007103.4 | c.1156C>T | p.Arg386Cys | Pathogenic | PS3_str; PM2_mod; PM3_str; PM5_sup; PP1_str; PP3_sup | Varghese et al 2015^15^; Ortega-Recalde et al 2013^16^ |
| *NDUFV1* | NM_007103.4 | c.1268C>T | p.Thr423Met | Likely Pathogenic | PS3_mod; PM2_mod; PM3_str; PP3_sup | Varghese et al 2015^15^ |
| *PDHA1* | NM_000284.4 | c.759+26G>A | p.Asp255Argfs*22 | Likely Pathogenic | PS3_mod; PM2_mod | Mine et al 2003^17^ |
| *PDHA1* | NM_000284.4 | c.506C>T | p.Ala169Val | Likely Pathogenic | PS3_str; PM2_mod; PP3_sup | Quintana et al 2010^18^ |
| *PDHX* | NM_003477.3 | c.1231C>T | p.Gln411* | Pathogenic | PVS1_vstr; PS3_sup; PM2_mod; PM3_sup | NOVEL VARIANT |
| *PDHX* | NM_003477.3 | c.1159C>T | p.Gln387* | Pathogenic | PVS1_vstr; PS3_mod; PM2_mod; PM3_mod | Brown 2006^19^ |
| *SLC19A3* | NM_025243.4 | c.1324_1327delinsAT | p.Tyr442Metfs*35 | Likely Pathogenic | PVS1_mod; PM2_mod; PM3_sup; PP4_sup | NOVEL VARIANT |
| *SUCLA2* | NM_003850.3 | c.434C>A | p.Thr145Lys | Likely Pathogenic | PM2_mod; PM3_mod; PP3_sup; PP4_sup | NOVEL VARIANT |
| *SUCLA2* | NM_003850.3 | c.272n.d.2A>C | splicing | Pathogenic | PVS1_vstr; PM2_mod; PM3_str | NOVEL VARIANT |
| *SUCLA2* | NM_003850.3 | c.1271del | p.Gly424Aspfs*18 | Pathogenic | PVS1_vstr; PM2_mod; PM3_mod | Carozzo et al 2015^20^ |
| *SUCLA2* | NM_003850.3 | c.1219C>T | p.Arg407Trp | Pathogenic | PS3_sup; PM2_mod; PM3_vstr; PP3_sup | Alkhater et al 2020^21^ |
| *SUCLA2* | NM_003850.3 | c.851G>A | p.Arg284His | Likely Pathogenic | PM2_mod; PM3_sup; PM5_mod; PP3_sup | NOVEL VARIANT |
| *SURF1* | NM_003172.4 | c.312n.d.321delinsAT | p.Leu105* | Pathogenic | PVS1_vstr; PM2_mod; PM3_vstr | Wedatilake et al 2013^22^ |
| *SURF1* | NM_003172.4 | c.515+5G>C | splicing | Likely Pathogenic | PM2_mod; PM3_mod; PP3_sup; PP4_sup | Wedatilake et al 2013^22^ |
| *SURF1* | NM_003172.4 | c.792_793delAG | p.Arg264Serfs*27 | Pathogenic | PVS1_vstr; PM2_mod; PM3_vstr | Lee et al 2012^23^ and Li et al 2018^24^ |
| *SURF1* | NM_003172.4 | c.574_575insCTGC | p.Arg192Profs*8 | Pathogenic | PVS1_vstr; PM2_mod; PM3_vstr | Lee et al 2012^23^ |
| *SURF1* | NM_003172.4 | c.752n.d.2A>G | splicing | Pathogenic | PVS1_vstr; PM2_mod; PM3_mod | Poyau et al 2000^25^ |
| *SURF1* | NM_003172.4 | c.488T>G | p.Val163Gly | VUS | PM2_mod; PP4_mod; BS4_sup | Clinical Leigh syndrome; ongoing in.house analysis |
| *TACO1* | NM_016360.4 | c.460T>C | p.Ser154Pro | Likely Pathogenic | PS3_sup; PM2_mod; PM3_sup; PP3_sup; PP4_sup | NOVEL VARIANT |

**Supplementary Table 1:** Classification of variant pathogenicity based on criteria from the ACGS 2020 guidelines

**Supplementary Table 2**

| **Gene** | **mtDNA (NC_012920.1) variant** | **Protein** | **Muscle heteroplasmy** | **Blood heteroplasmy** | **Fibroblast heteroplasmy** | **Urine heteroplasmy** |
| --- | --- | --- | --- | --- | --- | --- |
| *MT-ND1* | m.3688G>A | p.Ala128Thr | 84% | 5% | 19% | n.d. |
| *MT-ND4* | m.11778A>G | p.Arg340His | n.d. | 96% | n.d. | n.d. |
| *MT-ND4* | m.11778A>G | p.Arg340His | n.d. | Homoplasmic | n.d. | n.d. |
| *MT-ND4* | m.11778A>G | p.Arg340His | n.d. | Homoplasmic | n.d. | n.d. |
| *MT-ND5* | m.12706T>C | p.Phe124Leu | 64% | n.d. | n.d. | n.d. |
| *MT-ND5* | m.13513G>A | p.Asp393Asn | n.d. | n.d. | n.d. | 60% |
| *MT-ND6* | m.14459G>A | p.Ala72Val | 89% | n.d. | n.d. | n.d. |
| *MT-ATP6* | m.9176T>C | p.Leu217Pro | 98% | n.d. | n.d. | n.d. |
| *MT-ATP6* | m.9176T>C | p.Leu217Pro | n.d. | Homoplasmic | n.d. | n.d. |
| *MT-ATP6* | m.9176T>C | p.Leu217Pro | n.d. | Homoplasmic | n.d. | n.d. |
| *MT-ATP6* | m.8993T>G | p.Leu156Arg | n.d. | 95% | n.d. | n.d. |
| *MT-ATP6* | m.9035T>C | p.Leu170Pro | n.d. | 96% | n.d. | n.d. |
| *MT-ATP6* | m.9176T>C | p.Leu217Pro | n.d. | n.d. | 96% | n.d. |
| *MT-ATP6* | m.8993T>C | p.Leu156Pro | n.d. | 98% | n.d. | n.d. |
| *MT-ATP6* | m.8993T>G | p.Leu156Arg | n.d. | 91% | n.d. | n.d. |

**Supplementary Table 2:** Level of mtDNA variant heteroplasmy in available tissues. n.d. denotes not determined

**REFERENCES**

1. Ellard S, Baple EL, Berry I, et al. ACGS Best Practice Guidelines for Variant Classification 2020. 2020.

2. Pomponio RJ, Hymes J, Reynolds TR, et al. Mutations in the human biotinidase gene that cause profound biotinidase deficiency in symptomatic children: molecular, biochemical, and clinical analysis. Pediatric research. 1997;42(6):840-8.

3. Wiltink RC, Kruijshaar ME, van Minkelen R, et al. Neonatal screening for profound biotinidase deficiency in the Netherlands: consequences and considerations. European Journal of Human Genetics. 2016;24(10):1424-9.

4. Heidary G, Calderwood L, Cox GF, et al. Optic atrophy and a Leigh-like syndrome due to mutations in the c12orf65 gene: report of a novel mutation and review of the literature. Journal of Neuro-ophthalmology. 2014;34(1):39-43.

5. Scheper GC, Van Der Klok T, Van Andel RJ, et al. Mitochondrial aspartyl-tRNA synthetase deficiency causes leukoencephalopathy with brain stem and spinal cord involvement and lactate elevation. Nature genetics. 2007;39(4):534-9.

6. van Berge L, Hamilton EM, Linnankivi T, et al. Leukoencephalopathy with brainstem and spinal cord involvement and lactate elevation: clinical and genetic characterization and target for therapy. Brain. 2014 Apr;137(Pt 4):1019-29.

7. Stellingwerff MD, Figuccia S, Bellacchio E, et al. LBSL: case series and DARS2 variant analysis in early severe forms with unexpected presentations. Neurology Genetics. 2021;7(2).

8. Mahajan A, Constantinou J, Sidiropoulos C. ECHS1 deficiency-associated paroxysmal exercise-induced dyskinesias: case presentation and initial benefit of intervention. Journal of neurology. 2017;264(1):185-7.

9. Olgiati S, Skorvanek M, Quadri M, et al. Paroxysmal exercise‐induced dystonia within the phenotypic spectrum of ECHS1 deficiency. Movement Disorders. 2016;31(7):1041-8.

10. Haack TB, Jackson CB, Murayama K, et al. Deficiency of ECHS1 causes mitochondrial encephalopathy with cardiac involvement. Ann Clin Transl Neurol. 2015 May;2(5):492-509.

11. Tucker EJ, Hershman SG, Kohrer C, et al. Mutations in MTFMT underlie a human disorder of formylation causing impaired mitochondrial translation. Cell Metab. 2011 Sep 7;14(3):428-34.

12. Simon M, Richard EM, Wang X, et al. Mutations of human NARS2, encoding the mitochondrial asparaginyl-tRNA synthetase, cause nonsyndromic deafness and Leigh syndrome. PLoS Genet. 2015 Mar;11(3):e1005097.

13. Kohda M, Tokuzawa Y, Kishita Y, et al. A Comprehensive Genomic Analysis Reveals the Genetic Landscape of Mitochondrial Respiratory Chain Complex Deficiencies. PLoS Genet. 2016 Jan;12(1):e1005679.

14. Alston CL, Veling MT, Heidler J, et al. Pathogenic Bi-allelic Mutations in NDUFAF8 Cause Leigh Syndrome with an Isolated Complex I Deficiency. The American Journal of Human Genetics. 2020 2020/01/02/;106(1):92-101.

15. Varghese F, Atcheson E, Bridges HR, Hirst J. Characterization of clinically identified mutations in NDUFV1, the flavin-binding subunit of respiratory complex I, using a yeast model system. Human molecular genetics. 2015;24(22):6350-60.

16. Ortega-Recalde O, Fonseca DJ, Patiño LC, et al. A novel familial case of diffuse leukodystrophy related to NDUFV1 compound heterozygous mutations. Mitochondrion. 2013 Nov;13(6):749-54.

17. Miné M, Brivet M, Touati G, Grabowski P, Abitbol M, Marsac C. Splicing error in E1α pyruvate dehydrogenase mRNA caused by novel intronic mutation responsible for lactic acidosis and mental retardation. Journal of Biological Chemistry. 2003;278(14):11768-72.

18. Quintana E, Gort L, Busquets C, et al. Mutational study in the PDHA1 gene of 40 patients suspected of pyruvate dehydrogenase complex deficiency. Clinical genetics. 2010;77(5):474-82.

19. Brown R, Head R, Morris A, et al. Pyruvate dehydrogenase E3 binding protein (protein X) deficiency. Developmental medicine and child neurology. 2006;48(9):756-60.

20. Carrozzo R, Verrigni D, Rasmussen M, et al. Succinate-CoA ligase deficiency due to mutations in SUCLA2 and SUCLG1: phenotype and genotype correlations in 71 patients. Journal of inherited metabolic disease. 2016;39(2):243-52.

21. Alkhater RA, Ahonen S, Minassian BA. SUCLA2 Arg407Trp mutation can cause a nonprogressive movement disorder–deafness syndrome. Annals of Clinical and Translational Neurology. 2021;8(1):252-8.

22. Wedatilake Y, Brown RM, McFarland R, et al. SURF1 deficiency: a multi-centre natural history study. Orphanet J Rare Dis. 2013 Jul 5;8:96.

23. Lee IC, El‐Hattab AW, Wang J, et al. SURF1‐associated leigh syndrome: a case series and novel mutations. Human mutation. 2012;33(8):1192-200.

24. Li Y, Wen S, Li D, et al. SURF1 mutations in Chinese patients with Leigh syndrome: novel mutations, mutation spectrum, and the functional consequences. Gene. 2018;674:15-24.

25. Poyau A, Buchet K, Bouzidi MF, et al. Missense mutations in SURF1 associated with deficient cytochrome c oxidase assembly in Leigh syndrome patients. Human genetics. 2000;106(2):194-205.
